# Citizen science or scientific citizenship? Disentangling the uses of public engagement rhetoric in national research initiatives

**DOI:** 10.1186/s12910-016-0117-1

**Published:** 2016-06-04

**Authors:** J. Patrick Woolley, Michelle L. McGowan, Harriet J. A. Teare, Victoria Coathup, Jennifer R. Fishman, Richard A. Settersten, Sigrid Sterckx, Jane Kaye, Eric T. Juengst

**Affiliations:** University of Oxford, Harris Manchester College, Mansfield Road, Oxford, OX1 3TD UK; The Pennsylvania State University, 128B Willard Building, University Park, PA 16802 USA; Centre for Health, Law and Emerging Technologies, Nuffield Department of Population Health, University of Oxford, Ewert House, Ewert Place, Banbury Road, Oxford, OX2 7DD UK; McGill University, Social Studies of Medicine, 3647 Peel, Room 207, Montreal, Quebec H3A 1X1 Canada; School of Social and Behavioral Health Sciences, Oregon State University, Corvallis, Oregon, 123 Women’s Building, Corvallis, OR 97331-8577 USA; Department of Philosophy & Moral Sciences Ghent University, Blandijnberg 2, 9000 Gent, Belgium; 333 MacNider Hall, Campus Box 7240, University of North Carolina, Chapel Hill, 333S. Columbia Road, Chapel Hill, NC 27599-7240 USA

**Keywords:** Care.data, Citizenship, Citizen science, Crowdsourcing, Big data, National health research, Participant centric initiatives, Precision medicine, Public engagement, Recruitment

## Abstract

**Background:**

The language of “participant-driven research,” “crowdsourcing” and “citizen science” is increasingly being used to encourage the public to become involved in research ventures as both subjects and scientists. Originally, these labels were invoked by volunteer research efforts propelled by amateurs outside of traditional research institutions and aimed at appealing to those looking for more “democratic,” “patient-centric,” or “lay” alternatives to the professional science establishment. As mainstream translational biomedical research requires increasingly larger participant pools, however, corporate, academic and governmental research programs are embracing this populist rhetoric to encourage wider public participation.

**Discussion:**

We examine the ethical and social implications of this recruitment strategy. We begin by surveying examples of “citizen science” outside of biomedicine, as paradigmatic of the aspirations this democratizing rhetoric was originally meant to embody. Next, we discuss the ways these aspirations become articulated in the biomedical context, with a view to drawing out the multiple and potentially conflicting meanings of “public engagement” when citizens are also the subjects of the science. We then illustrate two uses of public engagement rhetoric to gain public support for national biomedical research efforts: its post-hoc use in the “care.data” project of the National Health Service in England, and its proactive uses in the “Precision Medicine Initiative” of the United States White House. These examples will serve as the basis for a normative analysis, discussing the potential ethical and social ramifications of this rhetoric.

**Summary:**

We pay particular attention to the implications of government strategies that cultivate the idea that members of the public have a civic duty to participate in government-sponsored research initiatives. We argue that such initiatives should draw from policy frameworks that support normative analysis of the role of citizenry. And, we conclude it is imperative to make visible and clear the full spectrum of meanings of “citizen science,” the contexts in which it is used, and its demands with respect to participation, engagement, and governance.

## Background: public roles in science

The promise of emerging scientific capacities to distill useful knowledge from “big data” is leading the scientific community to seek out more efficient ways to gather large numbers of empirical observations for analysis. For human health research, this means gathering more data about more humans. Nationally sponsored health research initiatives, like the “care.data” project in England or the “Precision Medicine Initiative” in the United States (US), wish to develop ways to enlist more members of the public to support this research need. In this paper, we explore strategies that contemporary national research initiatives are employing to encourage public cooperation and their ethical and social implications.

**Fig. 1:**
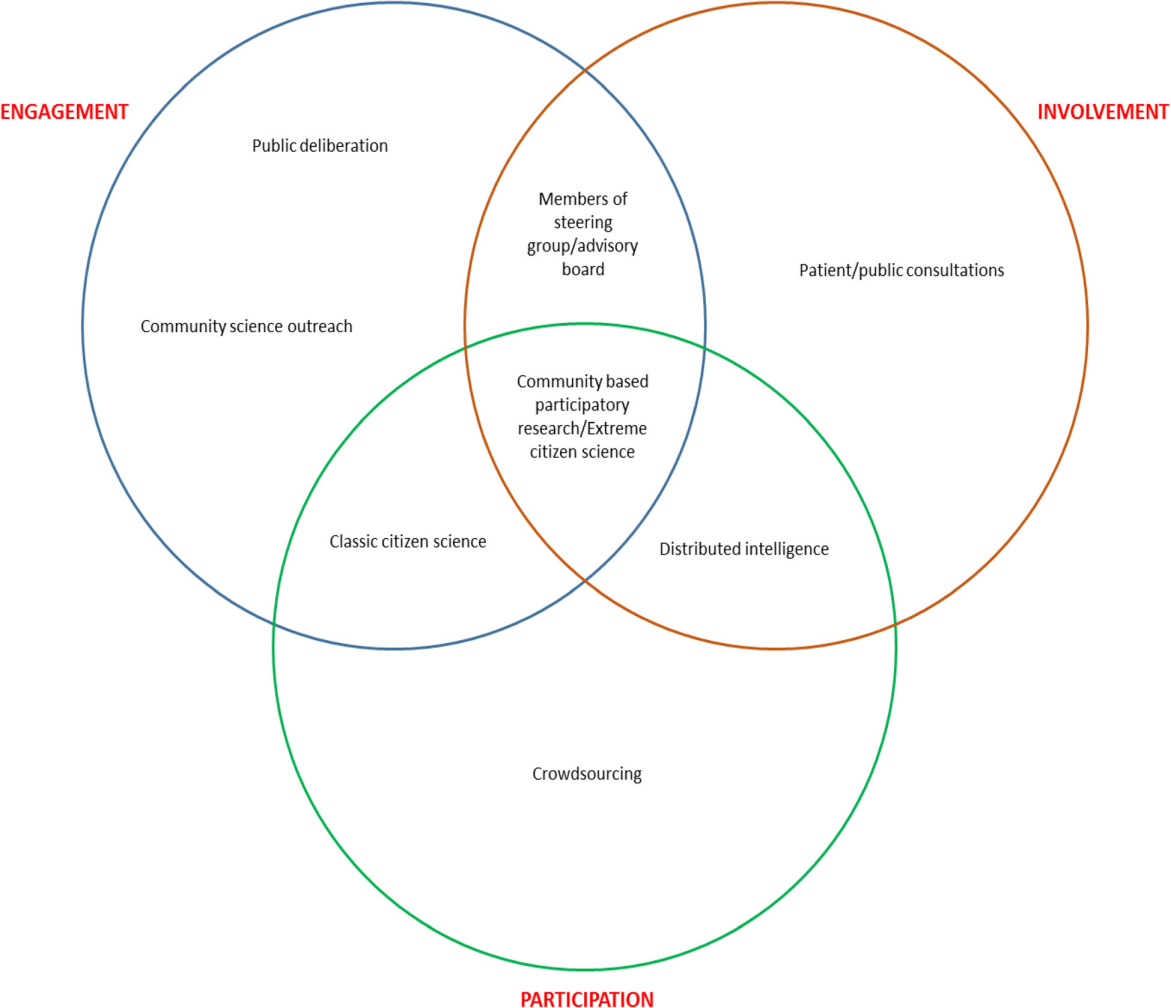
A useful way to depict the overlap between the three concepts of “participation,” “engagement,” and “involvement”

Members of the public become involved with scientific research in three distinct, if overlapping, ways. First, when the research is about some feature of their lives, the people’s role is to provide the data under study. Traditionally we describe this role as being human “subjects,” but today we often call it research *participation*. “Participation” suggests an active, intentional role, but can also describe quite passive forms of inclusion. People usually consent to participation in research about themselves; however, with proper safeguards and democratic approvals, the public might even be included in studies without their knowledge. Second, a common way to describe the public’s awareness and understanding of their participation, in turn, is in terms of their *engagement* with the research. Members of the public can be more or less engaged in scientific studies, depending on the extent to which scientists seek to communicate their plans and solicit the public’s cooperation in collecting data. In this sense, the public can be more or less ‘engaged’ with research whether or not they ‘participate’ in it because engagement is independent of their inclusion as research subjects. Third, in some cases, members of the public have an active role in in the planning and conduct of the research itself, even to the level of choosing the scientific questions to be addressed [1]. The UK National Health Service’s INVOLVE agency uses the term “*involvement*” to label this more active public role. A Venn diagram, which we present in Fig. 1, is a useful way to depict the overlap between the three concepts of “participation,” “engagement,” and “involvement,” [2].

At one level, the needs that national health research initiatives have for data about large numbers of people create a demand for more public participation. But unless the scientific community can justify involuntarily imposing such initiatives on the public, participation requires citizens to cooperate in making data about themselves available. “Engagement” and “involvement” practices are both ways to cultivate that cooperation by building public trust and a sense of collective investment in research. We are interested in the ways national health research initiatives invoke both types of practices in their promotional rhetoric and, in particular, their appeals to more active forms of citizen involvement in scientific governance. Specifically, we are interested in the ways these terms intersect with yet another term, “citizen science.” The citizen science movement of the last three decades has been a paradigm for active public involvement in science outside of health research. Recently, it has increasingly been used in health research as well. As “citizen science’ language penetrates more deeply into biomedical research, it raises questions about how “participation” should be interpreted, what normative factors are entailed by a given interpretation, and what this says about the rights, duties, and overall role of a citizen in biomedical research as various visions of societal good are pursued.

In this paper, we explore strategies that contemporary national research initiatives are employing to encourage public cooperation and their ethical and social implications. We begin with an analysis of the mixed uses of “participation,” “engagement,” and “involvement,” and how they pertain to “citizen science,” to bring normative questions surrounding this language to the fore. “Citizen science” offers a good starting place for our analysis, since the scientific appeal of its aspirations is relatively transparent. Below, we discuss a number of “citizen science” initiatives to demonstrate the wide range of efforts which employ the term. Next, we discuss the ways these aspirations become articulated in the biomedical context, with a view to drawing out the multiple and potentially conflicting meanings of “public engagement” when citizens are actually the subjects of the science. We then illustrate two uses of public engagement rhetoric to gain public support for national biomedical research efforts: its post-hoc use in the UK with the National Health Service (NHS) “care.data” project and its proactive use in the US White House “Precision Medicine Initiative.” These sections of our paper will then serve as the basis for a normative analysis, in which we probe the potential ethical and social ramifications of governmental use of this rhetoric to encourage participation in biomedical research. To foreshadow our conclusion, the ironic risk of this strategy may be that stronger public engagement rhetoric might be used to bolster a sense of civic duty to participate in national research initiatives, even when their benefits flow as much to the private sector as to public welfare. Looking ahead, new understandings of the social contract between science, government and the public may be required to address these health research risks to civil liberties.

### The growth and appeal of “citizen science”

“Citizen science” has become an umbrella term that applies to a wide range of activities that involve the public in science. It was coined independently by Rick Bonney in the US and Alan Irwin in the UK in the mid-1990s [3]. Bonney understood it primarily as a strategy for building public scientific literacy and trust, in which scientists and scientific institutions would take the “top-down” initiative to expose laypeople to the processes of scientific research in ways designed to cultivate their enthusiasm and support, [3] fitting in the public “engagement” category of Fig. 1. At a minimum, this could include educational efforts to increase public understanding and enthusiasm for science. However, the label could also apply to research projects enlisting large numbers of volunteer “amateur scientists” in the collection and reporting of data on factors in their surrounding environments. The citizens’ role, as volunteer data collection assistants, would be to contribute empirical observations for analysis by professional scientists. Under this interpretation, “citizen science” projects have been mounted toward wide-ranging objectives—from bird migration patterns and ecological trends to the identification of food security factors that act as early warning indicators for famine [4]. Beyond that, scientists also have begun to solicit the public’s help in data analysis—or at least in computer time—from NASA mission data to folding proteins and running large calculations [5]. At this level, the citizens might even claim to be “involved” in the conduct of science, albeit only as a lender of hardware and processing power. In all these cases, the goal is to propel science within the constraints of its traditional institutional contexts, and under the supervision of professional scientists [6].

By contrast, Irwin’s “bottom up” construction of citizen science is more policy directed, and describes practices more squarely in the “involvement” category of Fig. 1. For Irwin, “citizen science” has the goal of emancipating the pursuit of science from its traditional institutional and professional setting [7]. Under this interpretation, community-based urban planning or environmental science projects that are responsive to community needs and involve lay people in the conduct and governance of research are the exemplars of “citizen science.” These include lay environmental justice projects to examine the consequences of the Chernobyl nuclear accident [8] and Hurricane Katrina in Louisiana [9, 10]. At the extreme, lay people have begun using “crowdsourcing” and other techniques to conceive and conduct their own research projects outside of the traditional scientific community altogether [11].

When viewed through a normative lens, we see that Irwin and Bonney propose two different ways to advance the good of society. Bonney’s vision is based upon the presumed benefits of scientific knowledge in its own right. Irwin’s, in contrast, is based upon presumed emancipatory benefits of active public input on the direction of scientific research agendas. Bonney and Irwin’s two versions of “citizen science” are not necessarily mutually exclusive, but the difference in their orientation is reflected in the spectrum of initiatives with different objectives that have adopted the label, from projects that simply strive for public transparency to those that seek to put laypeople at the scientific controls. In any given initiative, the two visions of societal good may be aligned with one another. But when they do not align, it points to a fault line running through the conceptualization of the term “citizen science” itself—with contrasting visions of what constitutes societal good employing the same term to describe themselves. These differing visons for the role of scientific research in society call attention to the complex relationships between science, public goods, societal good, and public participation. They raise a key question: How much public input should go into deciding which vision of societal good a government should pursue?

The simple distinction drawn between Bonney and Irwin’s conceptualizations of citizen science highlights differences in how involving citizens in scientific research can advance societal good. When we look to the range of initiatives currently being described as “citizen science,” the picture gets much more complicated. Barbara Prainsack identifies nineteen parameters organised under six categories which allow for a much more granular and intricate understanding and classification of citizen science projects. This typology reveals just how complex and multifarious proposed relationships between science, public goods, societal good, and public participation can become [12].

Prainsack’s primary objective is to question whether or not citizen science changes how we assess expertise and authority in creating scientific knowledge, and how it does or should support participation in science. Yet a related aim is to determine whether and how citizen science makes science more socially robust by corresponding with dominant social, political, ecological, and commonly shared values. Or, if it makes science better in other ways, she asks, who benefits? [12]. Therefore, her typology could also be applied to help determine whether or not citizen science marks a change in how we assess expertise and authority in determining what vision for a better society to pursue, and how to best realise it given the complex interrelations discussed above. When seen in this way, Prainsack’s typology underscores just how many different conceptions of “societal good” could potentially be underlying citizen science initiatives.

However, despite these differences, across the spectrum, almost all citizen science initiatives share three features that make its rhetoric particularly appealing to apply to large population-based biomedical research projects: (1) connections to the spreading popularity of personal information communication technologies (ICTs); (2) “crowd-sourced” problem-solving; and (3) the “grass-roots” fundraising strategies that they facilitate.

First, the recent explosion of citizen science initiatives can largely be attributed to the integration of ICT into everyday life through computers, smartphones, the internet, and social media. This new potential for data collection provided by ICT is expanding opportunities for lay volunteers to collect, supply, and analyze information for data intensive research projects. Depending on the project, scores, hundreds, or thousands of volunteers can potentially provide millions of data points per month, [4] resulting in big data sets whose scientific significance is subsequently determined by experts and, in some cases, enhanced through statistical tools and techniques that facilitate analysis of complex data [13]. Citizen science is touted as a way to tackle otherwise intractable, laborious, and potentially costly research problems. Capable of coordinating the efforts of millions of lay people around the globe, it is said to allow researchers to think about data collection on a population-wide scale.

Second, the term “citizen science” is commonly used to not only refer to data collection, but also access to untapped resources that individual citizens can provide through ICT for data processing, interpretation, and problem solving. These “distributed intelligence” methods are said to offer scientists with low-cost options for data processing that could otherwise pose formidable barriers to research. A wide range of online interfaces and programs offered through, for instance, Zooniverse, [14] allow laypeople to assist with interpreting scientific datasets. These may provide images to the public in need of classification, ranging from galaxies (Einstein@home) [15] to tumour research (Cell Slider) [16]. Or, as with a Facebook-based game called Fraxinus, [17] they may be designed to allow the public to assist with sequence alignment in comparative genomics and pathogen genomics [18]. Or, as with Foldit, [19] they may be designed to enable the public to help determine the folding structure of proteins. In cases like these, “citizen science” becomes associated with various forms of technology-mediated social participation (TMSP), [20] where the level of participant engagement can vary from the passive loaning of a computers’ processing power, to a video game-based interface where there is little to no connection to the science, to an interface that requires genuine understanding of scientific principles. Although paradigms of crowdsourcing like Wikipedia are defined by the power of collective problem-solving, the literature that equates citizen science and crowdsourcing tends to present scenarios where citizens are only passively involved, having less to do with engaged citizenry or scientific literacy than with offering latent resources which, in one way or another, could advance a research agenda [21].

Finally, “citizen science” has been used in reference to fundraising for science, both philanthropically and politically. This usage also has a very high degree of overlap with crowdsourcing literature, in which citizen science is promoted as a means to improve funding applications through its ability to increase the impact of research by having an effect on the lay public. But more than this, there are also arguments for funding scientific research by petitioning individuals for financial support. Here, scientific research is presented less as a societal good and more as a way to provide products to potential investors. Citizen science initiatives are cast in terms of “excellent strategic investments for both private and government foundations,” as “critical for increasing public support,” and as something that can “open both pocketbooks and minds.” [22]. This presents scenarios where scientific goals, education goals, and financial goals vie for priority. There is a blurring of the distinction between educating the public about the value of science and “a sales pitch aimed at the public” in the marketing of a particular research agenda in that “[t]oday’s investors could become tomorrow’s advocates for scientific research,” [22]. This invites conjecture about the use of coercive lures, whether wealthy donors might expect special treatment, and whether research objectives might ultimately be correlated to financial generosity of certain individuals or groups over others [23].

The three core features of citizen science—leveraging of widely distributed ICT, harnessing the “collective wisdom” of the populace, and cultivating enthusiasm and support for science—make it very attractive to governments interested in propelling labor and data-intensive research in a cost efficient manner. For example, in the US, the Obama Administration’s 2013 Second Open Government National Action Plan [24] includes a “call to action” “for federal agencies to harness the ingenuity of the public by accelerating and scaling the use of open innovation methods such as citizen science and crowdsourcing in a variety of national priority areas,” [25] producing a wide variety of federally sponsored “citizen science” research programs under an overarching inter-agency “Community of Practice” initiative that seeks to “expand and improve the US government’s use of crowdsourcing, citizen science and similar public participation techniques for the purpose of enhancing agency mission, scientific, and societal outcomes,” [26]. These governmental initiatives lead to our focal question: What does “citizenship” have to do with “citizen science”? Two specific governmental initiatives, which we take up in section 3, are particularly instructive case studies: the English “care.data” project and the US “Precision Medicine Initiative.”

### Citizen science and biomedical research: creating a language of confusion

The vast majority of biomedical research takes place in the “participatory” sphere, where humans are the primary source of data gathered (whether those data are biometric, genetic, demographic, behavioral, etc.) *without* being “engaged” or “involved” beyond informed consent. However, recently there has been a turn to inflecting biomedical research (at least rhetorically) with “citizen science” aspirations, like those discussed above, where participants may have deeper investments in research. The language of involvement and engagement are used to entice and enlist these human participants to “opt-in” to research. Some examples of biomedical citizen science research projects include when patients have been asked to collect data on environmental factors on human health, monitoring severity of their own symptoms in space and time [27, 28]. Others have been asked to measure the microbes in their own guts [4]. In the case of the Personal Genome Project, the term “citizen science” has been used to describe a high profile genomics initiative where the “citizen’s” role is to provide the foundational data for any number of research agendas [23]. Participants agree to allow their genomes to become publicly available for multiple research objectives with variable risks. In this context, it has been argued that the meaning of “citizen science” has morphed to include community-based participatory research (CBPR), [23] in which affected communities participate in determining the scientific issues under study or partake in initiatives devoted to medical research on issues to which they are personally connected, such as the Coriell Personalized Medicine Collaborative. Subsequently, citizen science terminology has been extended to include DNA-related biotech hobbyism and direct-to-consumer genetic testing to produce a “genomics-informed citizen science” [23] and other forms of “participant-driven” or “patient-centric” genomic research [29]. One of the early proponents and catalytic enterprises to capitalize on citizen science ideologies was the privately-held, direct-to-consumer genetic testing company 23andMe.^1^ While 23andMe’s marketing focused on providing customers with personalized genetic information, [30] they also used the genomic and voluntary phenotypic data collected for genomic research, most infamously made evident by the patent they received in 2012 for polymorphisms associated with Parkinson’s Disease [31]. The “research” arm of the company, 23andMe, relied on citizen science and participatory research rhetoric, such as exhorting them to “join an effort to translate basic research into improved health care for everyone” in order to encourage its customers to become research “participants.” This included completing optional surveys about their health status, phenotypic traits, behavioral habits, and the like that could then be correlated with polymorphisms for traits of interest.

The aspiration to mobilize members of the public to conduct or take part in research has been particularly embraced by patient organizations that have identified a gap in the research agenda, and have sought to fill it themselves. Organizations such as Genetic Alliance, [29] Patientslikeme, [32] and genomera [33] acknowledge the expertise that patients bring to the research agenda, and yield considerable benefit from providing a framework in which patients are able to drive research, in some cases as an alternative to institution or government-led research agendas. Within biomedicine, a parallel description of projects can be found in “patient-centric initiatives” (PCI) or “patient-driven research” (PDR).

This overlap between PCI/PDR initiatives with other forms of “citizen science” has been previously recognized [34]. However, as section 1 demonstrated, the overlap only occurs at the more populist and proactive end of the spectrum of interpretations applicable to ‘citizen science.’ For a project to be classed as participant or patient-centric (or -driven), there is a fundamental requirement for patients or participants to play significant roles in identifying research priorities and helping to set the research agenda, as a “bottom up” enterprise in which lay people are not only engaged as learners and assistants, but also as scientists [35].

These terms vary with regard to the specific elements they encompass, how they fit with the research or healthcare agenda, the interpretation of the researcher or team applying them, and exactly what they mean for the patient or participant. For example, the use of the term ‘consumer’ as an alternative to either ‘citizen’ or ‘patient,’ carries a set of assumptions about the roles of individuals within a market-driven healthcare or biomedical research setting [36]. Empowerment, rights, and responsibilities are recurrent themes when presenting the implications of terminology for participation within a research project, with different terms seemingly selected to emphasize different aspects of these themes.

This is particularly noteworthy if the primary motivations for research groups to adopt engagement and involvement interventions include improving likelihood of funding, and subsequent recruitment of the required cohort [37]—which chimes directly with financial opportunity as a priority of citizen science, as identified in section 1. For instance, it is often the case that in order to improve participation it is necessary to engage potential recruits up front and throughout the research process. Involvement at certain stages throughout research development and implementation will also aid participation, by enabling targeted recruitment, and ensuring the research project meets the needs of its participants. However, in order to involve lay people and potential participants within this process, it will be necessary to engage with them first. It is for this reason that there is often confusion about exactly which part of this continuum is being addressed at any one time, and because engagement is perhaps the easiest and least involved, it is not unusual for engagement practices to be mistakenly described as involvement, to meet a requirement for involvement that has been imposed by a funding body or research institution.

Although there is confusion over what exactly the terms “involvement” and “engagement” cover, there are usually specific motivations for incorporating these elements into research projects which, in turn, dictate the specific tools used. Different citizen science projects purport to accommodate many of the features of interventions that could be described more generally as engagement, involvement or participation. However, the question arises as to whether the use of the general citizen science rhetoric poses a greater risk for confusion surrounding the goals of the research project and the expectations of different stakeholders, including participants, within it.

### Case studies

#### The care.data Project (England)

Confusion over different levels of citizen involvement can be particularly consequential when the rhetoric and practices of citizen engagement are used by national science initiatives to help rebuild public trust and support for projects that have already run into political trouble. One recent national biomedical science initiative that illustrates this defensive deployment is the “care.data” project in the UK.

In 2012, the UK Parliament passed the Health and Social Care Act (HSCA), which provides for the creation of the Health and Social Care Information Centre (HSCIC), a corporate body owned by the UK government with the power to collect, collate and provide access to the medical information for *all patients* treated by the NHS in England, whether in hospitals or by General Practitioners (GPs). Before the enactment of the HSCA, patients’ hospital data had already been collected and made available to researchers and others by the NHS Information Centre (set up by the Health and Social Care Act 2010), the forerunner to the HSCIC, and GPs had already started using standardised computerised record-keeping systems, but these records were not transferred to a central database.

The legal basis for the care.data project ‘trumps’ key provisions of the Data Protection Act [38]. The 2012 Act (the HSCA) allows all patient data to be used for purposes that extend beyond patient care (e.g. for research) without any consultation, i.e. without the patients’ knowledge. Thus, *the law makes it impossible for patients to prevent their data from being used for research*. Indeed, under the HSCA physicians are *obliged* to forward their patients’ medical records to the HSCIC which itself is *not obliged to inform the patient* of the use of such data once ‘anonymised.’ [38] 2

The NHS website states that it developed the care.data programme as an initiative “to ensure that there is more rounded information available to citizens, patients, clinicians, researchers and the people that plan health and care services,” and “to ensure that the best possible evidence is available to improve the quality of care for all,” [39]. Benefits of the scheme mentioned include the possibility for researchers to “identify patterns in disease and the most effective treatments”; the possibility “to find more effective ways of preventing or managing illnesses; advise local decision makers how best to meet the needs of local communities; promote public health by monitoring risks of disease spread; map out pathways of care to streamline inefficiencies and reduce waiting times; determine how to use NHS resources most fairly and efficiently,” As the healthcare provider for all UK residents, the NHS possesses an immense quantity of genotypic and phenotypic data, and has access to millions of patients’ bodies and tissue samples. Indeed, the NHS data bank is a potential goldmine for a range of researchers, including for-profit companies seeking to develop drugs or diagnostic tests.

The potential positive impact of the care.data programme has been acknowledged by various organisations, such as the Royal College of General Practitioners and the British Medical Association [40]. However, the scheme has met with significant opposition. In autumn 2013, NHS England had set up a care.data website where citizens could record their concerns. The public response was vocal, and included strong concerns about the following: lack of transparency; lack of respect for confidentiality and privacy; misgivings about the opt-out basis for participation; 3 erosion of trust in GPs and the health care system; wrongful appropriation of personal property; commercialisation; and uses of personal health data that conflict with the person’s moral values. One of the particular bones of contention was the prospect that commercial companies could have gained access to patient data through care.data, and that this was not communicated clearly to patients [41].

The government decided to delay GP data harvesting until autumn 2014 to allow NHS England the opportunity to persuade GPs, healthcare workers and patients that the care.data scheme was valuable and that sufficient safeguards had been put in place. It was decided to pilot the GP data harvesting within a group of ‘pathfinder’ areas in England, 4 and to initiate a series of public discussion sessions with the care.data Advisory Group. According to the most recent update provided by the British Medical Association: “The purpose of the pathfinder stage is to trial, test and evaluate the data collection process and communications to patients,” [42]. As a part of this pilot stage, the project sponsored 150 “listening events” for health professionals, patient groups and the public, during which “more than 3000 people shared their comments and concerns,” [43]. In announcing these engagement exercises, Tim Kelsey, then NHS England’s National Director for Patients and Information, explained that:

Care.data [is] a programme of work which aims to consistently and systematically use and join up data across hospitals and general practice *and make it available to the people who can use it to make services better—clinicians, commissioners, researchers, charities, patients and public****—***in safe ways that minimize the risk to a person’s privacy being compromised in an age of increasingly sophisticated digital threats…. *Data sharing between professionals, patients and public is the precondition for a modern, sustainable health and care service* (emphasis added) [44].

Calls for data-sharing between the professionals and their institutions in health care –the clinicians, commissioners, researchers, charities and, presumably, the commercial concerns dedicated to translating data into health care products—are nothing new. However, including patients and the public amongst those “who can use [data] to make services better” seems to promise a level of public engagement that verges on “involvement.” The architects of the care.data project came to this rhetoric primarily in response to a public outcry, however, and it remains unclear what role it might actually be promising, given traditional understandings of the translational research process.

#### The precision medicine initiative (US)

The framing of our second case study—the US government-sponsored Precision Medicine Initiative (PMI)—relies on the popularity of citizen science rhetoric. The PMI was announced by President Barack Obama in his State of the Union Address in January 2015 and reinforced by the leadership of Francis Collins, the director of the US National Institutes of Health (NIH), and by the infrastructure and resources of NIH [45].

The purpose of the PMI is to promote and pursue the “paradigm shift” that the Human Genome Project (HGP) promised for healthcare. As the White House website explains:

Until now, most medical treatments have been designed for the “average patient,” As a result of this “one-size-fits-all” approach, treatments can be very successful for some patients but not for others. Precision Medicine, on the other hand, is an innovative approach that takes into account individual differences in people’s genes, environments, and lifestyles. It gives medical professionals the resources they need to target the specific treatments of the illnesses we encounter, further develops our scientific and medical research, and keeps our families healthier [45].

The PMI consists of an infusion of US $215 million into efforts to accomplish this goal over a “3-4” year period, including “ US $130 million to NIH for development of a voluntary national research cohort of a million or more volunteers to propel our understanding of health and disease and set the foundation for a new way of doing research through engaged participants and open, responsible data sharing,” [46]. To accomplish this, Collins, writing with former director of the National Cancer Institute Harold Varmus, outlined the potential of precision medicine:

We envisage assembling over time a longitudinal “cohort” of 1 million or more Americans who have volunteered to participate in research. Participants will be asked to give consent for extensive characterization of biologic specimens (cell populations, proteins, metabolites, RNA, and DNA — including whole-genome sequencing, when costs permit) and behavioral data, all linked to their electronic health records [46, 47].

The government’s strategy has been to invoke the appeal of participant-driven “citizen science” to garner support for patient participation in the PMI, despite the irony of appropriating rhetoric intended to promote deinstitutionalizing and “democratizing” scientific research to increase support for an exercise of Executive Branch power through the government’s centralized biomedical research funding agency. As the White House reports:

Participants will be involved in the design of the Initiative and will have the opportunity to contribute diverse sources of data—including medical records; profiles of the patient’s genes, metabolites (chemical makeup), and microorganisms in and on the body; environmental and lifestyle data; patient-generated information; and personal device and sensor data. Privacy will be rigorously protected. This ambitious project will leverage existing research and clinical networks and build on innovative research models that *enable patients to be active participants and partners* (emphasis added) [46].

Since January 2015, a series of workshops and conferences have been held to flesh out this “patient-powered” vision of the PMI. These meetings have brought together architects of online crowd-sourced science projects, disease advocacy groups, and “consumer genomics” companies to extract lessons about participant-driven research [26].

One of the goals—and challenges—that has emerged in these conversations is how to create a “culture shift” such that patients and their families become “the true engines” of precision medicine [48]. To date, much of the foundation for this culture shift has been laid by parents whose children have rare or undiagnosed conditions: “Racing against the clock to save their children, parents are building databanks, connecting scientific dots and fueling therapeutic advances that could otherwise take a decade or more to happen,” [48]. As the leader of nonprofit health advocacy group Genetic Alliance effused, “this is what [we have] been working towards for years. I am delighted by the President’s recognition of the power of participants in this initiative. Together with other advocates, citizen scientists, and research participants, we are thrilled to roll up our sleeves and get started!” [49].

Isaac Kohane, who is chair of Harvard Medical School’s Department of Biomedical Informatics and hosted one of the conferences, similarly heralded the potential of a much larger culture shift such that patients “feel empowered morally and intellectually to lead in precision medicine research and delivery.” [48].

NIH also convened a PMI Working Group to develop an operational recruitment proposal for the PMI-Cohort Program of a million participants [27]. When it issued its recommendations in September 2015, the NIH PMI Working Group report embraced this culture shift explicitly: “Participant engagement and empowerment are core values for the PMI-Cohort Program (PMI-CP). Whereas the majority of clinical research has been transactional in nature, with unidirectional data sharing from the individual to the study, the PMI-CP seeks *true partnership between participants and researchers”* (emphasis added) [50].

Building that partnership, the Working Group goes on to say, relies on better communication with the public so that individuals understand the significance of research and how their participation in it brings health benefits for themselves, their families, and their communities. A primary goal of the PMI cohort is therefore to “empower individuals to understand potential opportunities to manage their health offered through genomic sequencing, aggregation of longitudinal health information, and sharing of data with researchers, under a *cooperative model of partnership and trust* … and *shared responsibility* for health knowledge (emphasis added),” The Working Group expects the PMI to “exemplify engagement at its best,” [50]. The PMI cohort, as it is built, will require a “substantial variety of data access and analysis services and support to help researchers of varying levels of sophistication, including ‘citizen scientists’ and study participants, achieve their research goals.” [50].

Although the term “citizen science” is not regularly used in the PMI initiative documents, it is clear that the White House is actively trying to promote the spirit of “open science and innovation,” values that are core to participant-driven citizen science initiatives [11]. A recent White House webcast was designed to raise awareness of citizen science and crowdsourcing tools as “approaches that educate, engage, and empower the public to apply their curiosity and talents to a wide range of real-world problems,” and ultimately build a science “of the people, by the people, for the people.” [19]. To this end, Francis Collins wrote:

[T]he Precision Medicine Initiative Cohort Program will change the way we do research. Participants will be partners in research, *not subjects*, and will have access to a wide range of study results. What we’re doing with the Precision Medicine Initiative cohort is intersecting in a synergistic way with other fundamental changes in medicine and research to empower Americans to live healthier lives (emphasis added) [51].

In considering the case study of the PMI it is important to note that this initiative is still unfolding, having been announced at the beginning of 2015. The ramifications of utilizing rhetorical appeals to citizen science in framing opportunities to participate in a national research initiative will need to be traced as recruitment, enrollment, and involvement of participants into the PMI-CP gets underway.

## Discussion: Ethical and policy implications

It is true that the sociopolitical contexts of care.data and the Precision Medicine Initiative are not entirely alike. Dissimilar healthcare systems in the UK and the US can lead to different views on the role of the government in the administration of population-based medical research. For instance, in the UK, where healthcare is seen as a right afforded to all citizens, it is natural to expect that government institutions like the NHS will hold health-related data for the purpose of treating individuals and populations. It is expected that the government, and not external third parties, will be responsible for ethical stewardship of that data. In the US, on the other hand, where private healthcare is the norm, this is not the case. Here, private institutions are responsible for stewardship. Government involvement occurs, more often than not, through regulatory restrictions on how data should be managed and whether it should be shared beyond a given institution. The tenor of the former is more proactive, the tenor of the latter is more reactive. This distinction marks a key difference which reverberates throughout the two case studies. Yet when the connection between an individual’s data and an individual’s care is severed so that data is shared or sold with the more general aim of improving biomedical research, the relationship between the individual and the institution trusted with stewardship is significantly altered. In this way, the two cases are alike. And they each raise normative questions about the role of government in managing the interests of the individual and the interests of others who would benefit from, or stand to profit from, access to that individual’s data.^5^

Both the English care.data project and the US Precision Medicine Initiative are research projects initiated by national governments to address health care questions by using biomedical information about their citizens. As such, these governments put their respective populaces in the position of being offered for study by academic scientists, health care institutions, and private industry. Unlike the national census, neither initiative is critical to either nation’s ability to govern. Moreover, unlike population health surveillance efforts like the US Center for Disease Control’s “National Health and Nutrition Evaluation Survey,” neither initiative is primarily aimed at governmental planning for national public health needs. Instead, both are aimed at improving the medical interventions that their health care systems can offer patients—by increasing the amount and variety of biomedical data to which non-governmental scientists, entrepreneurs, and commercial entities have access in order to develop new medical interventions.

The governmental leveraging of such “public/private partnerships,” allegedly for the benefit of public welfare and national economies, is not unusual. And, although both initiatives were conceived and launched by executive rather than representative branches of government, they were eventually vetted and authorized by each country’s political process. However, the roles of the citizenry in each of our case studies are quite different, and they shed cautionary light on how the popular appeal of “citizen science” and “public engagement” rhetoric can be (mis)used in state-sponsored biomedical research, even in liberal democratic nations.

Many unanswered questions lurk beneath this rhetoric. Complicating factors include the ways various interests are represented in government; the ways potentially conflicting interests collide in mandated data sharing; the influences of commercial interests over and above individual interests in policy development; and the inadequate attention given to the implications of the roles that the citizenry plays. We discuss each of these in turn.

With respect to the potential for conflicting interests colliding in mandated data sharing, the U.K. care.data project presents itself as a “data-sharing” project rather than a “citizen science” or “public engagement” project *per se*. “Data-sharing” is a label that typically describes a professional transaction between scientists—the practice of pooling or combining data from separate investigations to provide a more robust foundation for research—and has gained wide appeal within the scientific community over the last two decades as an antidote to an increasingly competitive and secretive research environment credited to the advent of commercial interests in the life sciences [52]. In that context, “data sharing” in science resonates well with the appeal of “open source” software development and collective “crowdsourced” resources like Wikipedia. Initially, this provided a persuasive platform for the launch of care.data, from which its proponents could argue that the NHS’s individual patient confidentiality concerns impeded progress [53].

Contemporary science policy emphasizes the need to use “technology transfer” from the public to the private sector to translate basic science “from the bench to the bedside,” Most data-sharing initiatives, including care.data, are as much about speeding the commercialization of research findings as they are about open communication between clinicians and/or scientists. It is a case of the asymmetric influence of commercial interests over and above individual interests in policy development. Indeed, the prospect of (re)identifiable personal medical information flowing from the NHS into the private sector without patient permission is what provoked the groundswell of public concern that has delayed the initiative and the eruption of “public and patient engagement” rhetoric to show how “NHS is committed to listening to and working with professionals and patients to gather their feedback on how to implement care.data.” [53, 54].

Expanding the circle of “the people who can use [shared Electronic Medical Records data] to make services better” to include patients and the public, and citing their involvement in data-sharing as a “precondition for a modern, sustainable, health and care service,” [44] could be read as a remarkable commitment of NHS to the democratization of biomedical research and public empowerment, very much in the spirit of “citizen science” in its robust sense. It suggests that patients and the public will have direct access to the data collected through the care.data project and be given roles in the research process.

Or does it? Despite this rhetoric, the care.data project’s rules are vague on what kinds of third parties may purchase access to the data, and it is not clear whether the project’s “listening events” have had any impact on its scientific design or research mission. Instead, what the NHS seems to mean by “data sharing between professionals, patients and the public” as a fundamental precondition for its service is simply “data sharing between professionals,” ideally with patient and public understanding. “Informational altruism” may be a modern day civic virtue, but until we reach the point at which medical confidentiality endangers the social fabric, a willingness to share one’s self with the world remains a virtue to be praised rather than encouraged through insinuations of co-ownership and control.

These attempts to engage the public do not draw from policy frameworks that support normative analysis of the role of citizenry in new and emerging forms of governance. Rather, care.data provides an example of a governmental initiative, in response to public reactions, defensively backing into public engagement rhetoric. What began as a “social license” to gather and curate health data begins to turn into a claim about good citizenship [41]. In underscoring the public’s civic duty to participate in biomedical research, [55] it tries to provide a strong enough justification for the conscription of private medical information and its dissemination outside of the national health care system.

Concerns from professional medical bodies and citizens about the implementation of care.data illustrate the challenges of finding balance between aiming to improve the quality of care and health services (and stimulate research) on one hand, and respecting ethical values such as trust, autonomy, transparency, and confidentiality and privacy on the other. The general public is generally positive towards medical research and is usually willing to participate without expecting any personal benefit [56, 57]. People are less willing to participate, however, if the benefits to society are unclear or if private profits might be derived [58]. In order to merit and garner trust, guardians of citizens’ health data ought to ensure that they respect the values of the people who are expected to trust them with their data. Citizens should not have any fear that they are being manipulated into sharing their health data.

In the US, the PMI has been explicit in its appeal to populist rhetoric from its inception. One question this leads to is whether its appeals are any more substantive than those of care.data. If the PMI is sincere about giving its participants a substantive role as “partners” in its investigations, a second question follows: What should we make of that from an ethical perspective? For example, does it make a difference ethically whether the public participants are merely data collection assistants or genuine “co-investigators”?

The vast majority of the PMI samples and data will be gathered through participating biobanks and health care organizations, which will add a layer of communication complexities and informational barriers between individual sources and the PMI-CP at NIH. Even with explicit “opt-in” procedures in place for patients and biobank contributors, it is not clear whether these participants will have any more direct engagement with the PMI than the patients whose medical records are opened in the care.data initiative. With proposed changes to the US human subjects research regulations, [59] patients could “consent” to their sample and data donations as routine paperwork upon admission to the hospital, and be justifiably surprised when their first PMI participant newsletter arrives in their (e)mail.

At the same time, for those who proactively enroll as “participants at large,” it does seem clear that the PMI would like to take advantage of the growing interest in personal health monitoring to enlist their services as data collectors for the PMI and its scientific and commercial clients [50]. In this sense, the PMI displays the same weak form of “citizen science” celebrated by other governmental and institutional initiatives that enlist members of the public to collect and report empirical observations, such as the UK’s OPAL projects [60]. As (unpaid) scientific field-workers, participants are invited to donate their health monitoring time and energy to the initiative and, in return, are promised regular reports of the initiative’s progress. Unlike citizen science projects in astronomy, ornithology, or ecology, which depend on the assistance of engaged members of the public, the PMI instead promises tangible “translational” benefits that could be shared: both health benefits for families suffering from rare or recalcitrant diseases, and financial profits to be made by turning its findings into medical innovations. Both of these forms of translational benefits raise the stakes of contributions to the PMI enough to make the participants’ informational altruism look less like laudable civic volunteerism and more like the exploitation of the gullible.

The partnership rhetoric of the PMI, however, does seem to offer a more substantive role for its public participants. But what could that role be? There is little chance that participants will be encouraged to conduct “DIY genomics” studies of their own using the PMI’s platform, or that “the ingenuity of the American people” will be harnessed through “crowdsourcing” to solve scientific puzzles. Despite its references to personal empowerment and the interests of rare disease advocacy groups in organizing their own research efforts for the benefit of their constituencies, the PMI is not proposing to make citizens scientific co-authors of its research in either literal or figurative ways. At the same time, the PMI does seem to offer its participants some role in the governance of the initiative. Perhaps this is the sense in which it aspires to rise to the level of “citizen-led” science.

The implementation plans for the PMI are still being developed, so it is difficult to discern how the project’s projected one million biospecimen and data donors will be meaningfully engaged as “partners.” The donors who will make up the bulk of the collection are unlikely to be actively engaged in design and management because its infrastructure and processes will need to be in place before they are recruited. It is possible that the PMI’s public “participants at large” will be more motivated to be engaged, but how their voices will be heard is unclear, short of recreating a democratic system of elections from within the cohort for representation on the PMI’s governing committees. Already, some of the constituencies that might naturally represent segments of the cohort, like advocacy groups and charities, have been cut out of the governance system in a move to avoid the influence of “special interests,” [50].

The populist rhetoric of the PMI is clearly at least in part a strategic effort to increase public participation and support. Governmental agencies and Big Science projects need ever-larger numbers of people to pursue their population genomic goals, and they are happy to trade on the ideals of solidarity and volunteerism that accompany “citizen science” to meet those needs. As such, populist rhetoric appeals to the rising public interest in participant-driven disease advocacy research and citizen science contexts, which is also often accompanied by frustration with the bureaucratic constraints of the research regulatory process [11]. Ironically, in the PMI context, the reforms that proponents advocate are not designed to deinstitutionalize research or make it easier for lay people to control. Instead, they are aimed at making it easier for research institutions to advance the movement’s medical and political goals by diluting and dismantling conventional individual informed consent requirements. Such a top-down approach to citizen science runs the risk of watering down the role of the citizen research subjects in the PMI’s “genomic research participant corps” to passive sources of data [11].

Perhaps in recognition of this potential, advocates of the PMI are anxious to expose and address the problem that the biomedical research regulatory system in the US is not set up to facilitate de-institutionalized “citizen science” efforts. As Collins and Varmus advocate in their manifesto for the PMI:

Achieving the goals of precision medicine will also require advancing the nation’s regulatory frameworks. To unleash the power of people to participate in research in innovative ways, the NIH is working with the Department of Health and Human Services to bring the Common Rule, a decades-old rule originally designed to protect research participants, more in line with participants’ desire to be active partners in modern science [47].

In this regard, it is telling that, despite its invocation of the populist appeal of “bottom up” citizen science, the White House uses a naval metaphor to promote the PMI, urging that: “[t]ranslating these successes to a larger scale will require a national effort: to push this effort forward we will need all hands on deck, including patients, hospitals, industry,” [61] This suggests that members of the public, and the research institutions with which they interact, are part of one national crew, susceptible of being called to duty as required by the needs of the ship of state. Taken to its logical conclusion, this line of thinking could even be used to support an expectation that every citizen become engaged in precision medicine research, as a matter of collective duty, running the risk of re-inscribing prejudicial ascribed identities with the very scientific tools that could be used to bring us past them to a real “paradigm shift” for biomedicine, and the use of those identities to unfairly influence participation in research and public health interventions [62].

The traditional, localized, case-specific contexts around which much policy development formed over the past 50 years has not prepared us for the regulatory challenges we now face. Biomedical research has been based upon a model of altruistic gift giving of sample and data to generate knowledge in the service of promoting the greater good of society. But now that model is being used to support an entirely different research edifice, which elicits entirely different normative concerns. The term “partnership” is misrepresentative. It masks a complex state of affairs in which the aggregation of diverse interests that result from data sharing is met with an uncomfortable (and greater) influence of government. This asymmetry raises the specter of governmental and commercial interests that impinge on individual rights, and renders nominal at best any claims of advancing societal good. What is to keep a self-serving model of industry from destroying the public trust and good faith that the biomedical community has cultivated over the past half century? Even if a vast majority of commercial interests maintain a significant convergence with the good of society, it does not take many bad actors to poison the well.

The concept of “partnership” implies not only co-leadership but co-ownership, with an equitable share in a collaborative venture’s returns. Promising that the PMI will rise to the level of a co-production between the PMI-CP, its scientific and commercial clients, and its public participants suggests that those participants might reasonably see their contributions not as altruistic donations but as investments—and expect to reap tangible dividends, either in terms of health care benefits or financial profits. Inevitably, this sense of public solidarity that the PMI seeks to cultivate evaporates under the pressure of this logic, as groups of participants find common ground in trying to influence the initiative’s governance to prioritize their own special interests.

Ironically, the UK and the US already struggle with this same issues of engagement, involvement, and participation in their efforts to mount international genomic research collaborations like the “H3 Africa” initiative. Just as the nations participating in those efforts seek to protect their own interests by declaring “genomic sovereignty” over their populations’ biospecimens and data, it will be reasonable for families (like the descendants of Henrietta Lacks) to seek to protect their “genetic legacies” from exploitation, rare disease groups (like PXE Int’l) to secure patent protections in the commercialization of “their genes,” and communities (like the Havasupai tribe) [63] to demand control over the questions scientists might pursue using “their people’s” samples [64].

## Conclusion: taking “citizen” seriously

In considering the policy implications of our analysis, it is helpful to return to the phrase “citizen science,” The current facile use of “citizen” in “citizen science” in the biomedical community conceals a real need. If the term “citizen science” is to be widely used, policy concerning it needs to be in tune with the normative realities of biomedical data sharing. In employing the term “citizen,” “citizen science” initiatives fuse the languages of bioethics and governance. Consequently, in addition to raising questions concerning ethical oversight of medical research, they also raise questions concerning just participation and engagement. They call into question what the reciprocal relationship between and individual and society ought to be. This question has been a focus of bioethicists who have called for a new social contract between individuals and society that can better support biomedical research [65–69]. There is disagreement about what a new social contact should include. But, nevertheless, a model of participation and engagement which draws on the idea of a social contract would allow one to more readily think beyond current models, such as the altruistic and profit-oriented ones discussed above, which can easily conflict. It would allow one to think more directly about what being a citizen in a just society means.

It is clear that many who use the term “citizen science” have quite different views of what the term “citizen” means. This has ramifications for their views on what “societal good” means, on individual rights and duties, and on what authority governments should possess. Each perspective therefore carries with it its own set of normative assumptions and consequences. At its most immediate level, the use of the word “citizen” lays emphasis on the relationship between individuals and the societies within which they live. It naturally raises the question: What is the relationship between citizen responsibilities and government regulations when viewed against the societal good that biomedical sciences promises to provide? While the question is simple, the answer is, of course, complicated. However, some points are less contestable than others. For instance, in democratic societies, it is not too controversial to say that “citizen” is inextricably linked to some form of self-determination; autonomy helps justify a government’s authority. Citizens may thereby hold the protection of autonomy sacrosanct.

In government led initiatives like care.data and PMI we face more than the perennial bioethical challenge of balancing the greater good with individual rights. Above, we mentioned Prainsack’s typology as one way to better understand competing visions of societal good by determining whether and how a given citizen science initiative makes science more socially robust by corresponding with commonly shared values. In democratic societies, the preservation of autonomy is one of those commonly shared values. Therefore, if a citizen science initiative is to be portrayed as both socially robust and as advancing societal good in a democratic society, public participation cannot be decoupled from the process that determines the vision for society being pursued. Further, as autonomy is also a primary principle of bioethics and related law, there is an essential nexus of normative principles that span both governance and bioethics such that individual interests need to be respected and harmonized along the lines of sound, clearly articulated, principles which present a coherent vision of the rights and duties of, not just citizens, but of all actors. In the context of biomedical research, autonomy is most directly expressed by the giving of consent. In this sense, a strong connection can be drawn between the roles of consent in political theory, in bioethics, and in the pursuit of societal good in democratic societies. 6

As we have seen, in citizen science contexts, ICT offers new models of data collection, processing and analysis, and thus new kinds of opportunities for engagement in these aspects of research. One of the key support mechanisms for participation is the consent process. Depending on the participatory model and the specific research project, the expectations of the role of the participant can lead to variation in the approach to consent. Such variation can be a challenge for policy development. Yet, ICT also present new opportunities for governance of research, specifically for consent, which help to address this challenge. For example, dynamic consent [70] is being explored as an opportunity for participants to have greater, more nuanced control over their participation in research by being able to determine how their data are used, and even to change their mind as research progresses. It could also be used as a tool during research, to update participants on the progress of research [71]. This could greatly encourage participation and engagement. Some consider the use of interactive information technology like these that engage and communicate with participants as central to advancing PCIs [70]. Such a tool would transform the role of the participant in “citizen science” initiatives, and ensure that the giving of consent remains linked to concepts of citizenry. 7

Hence, when the term “citizen” is taken seriously, we can better consider the various contexts within which “citizen science” is used and the ethical significance of differences in its uses. This enables us to better see what vison of societal good is being advanced by the research, and what other visions are, perhaps, being sidelined by it. This allows us to be more cognizant of which ethical ideals are characteristics of which initiatives. It allows us to see specifically why the ideals of one initiative are not necessarily compatible with those of others. We then can better appreciate the problems created for and by researchers and policymakers when these differing ideals are represented by a single term, which wrongly suggests a common ethos.

It is therefore imperative to make visible and clear the full spectrum of meanings of “citizen science,” the contexts in which it is used, and its demands with respect to participation, engagement, and governance. For instance, in the examples discussed above, the principle of autonomy is approached quite differently. In care.data, whether the government should have the power to conscript its citizens for their data becomes an important question. But in participant-centric research initiatives, empowerment of the individual is central. The balance struck between individual autonomy and a greater societal good is different in each case.

Similarly, the relationship between the citizen and the powers of government is conceived quite differently in citizen science initiatives that tout antiregulatory ideals compared to those that seek to create regulatory infrastructure. The very idea of “governance” is polarizing. Differences like these are fairly obvious. But the fact that they can all potentially be billed as “citizen science,” with no reference to what “citizen” means or what normative principles are involved, muddles communication.

As was made evident in the discussion above, broaching this topic promises to create veritable storm of controversy and debate on the very things that facile, uncritical usage of “citizen science” fails to address. The eye of this storm is the changing requirements for consent and, by extension, the changing contexts in which the principle of autonomy is to be applied, or not, as governance develops. As such, it provides a natural focal point for deliberation.

There is, of course, already ample debate on what the role of consent should be in the many contexts of biomedical research. But these are largely framed within, or in reaction to, existing bioethical or policy frameworks and oversight mechanisms. The approach proposed here is different. Explicating the varied meanings and uses of “citizen” will in each case also eventually reveal what role is envisioned for participation and engagement. Thus we can articulate more precisely what distinguishes one “citizen science” initiative from another specifically in terms of principles of governance.

In thinking holistically about participation, engagement and governance, one can further consider what bearing each “citizen science” initiative has on a citizen’s rights and duties, on definitions of “societal good,” on the powers of government, and on which interests should hold sway over others. Disambiguating what “citizen science” means along these parameters, one can more methodically consider what among these many elements gets priority, what gets minimized, and why—in other words, one can articulate what one thinks the new social contract should be. This new framing allow us to cut through the layers of complications discussed above: the ways various interests are represented in government; the ways potentially conflicting interests collide in mandated data sharing; and the asymmetric influences of commercial interests over and above individual interests in policy development. Ultimately, as attempts are made to engage the public, this modelling will help to address the disconnect that occurs between government and the citizenry—by providing a policy framework that allows for normative analysis of the role of citizenry in new and emerging forms of governance.

## Summary

In this paper, we explored ethical and social implications of strategies that contemporary national research initiatives employ to encourage public cooperation. We began with an analysis of the mixed uses of “participation,” “engagement,” and “involvement,” and how they pertain to “citizen science.” And we showed that the multiply ways “citizen science” is used creates scenarios where scientific goals, education goals, and financial goals vie for priority.

We then examined a recent turn to inflect biomedical research with “citizen science” aspirations, suggesting participants may have deeper investments in research. Noting the significant confusion that already exists over the ways the terms “participation,” “involvement” and “engagement” are used, we argued general citizen science rhetoric poses even a greater risk for confusion. Confusion over different levels of citizen involvement can be particularly consequential when the rhetoric and practices of citizen engagement are used by national science initiatives that address health care questions by using biomedical information about their own citizens. We looked at two such initiatives, the UK care.data and the US Precision Medicine Initiative.The architects of the care.data project came to this rhetoric primarily in response to a public outcry. It remains unclear what role it might actually be promising to UK citizens. The framing of PMI relies on the popularity of citizen science rhetoric, and yet the type of “partnership” is proposes with participants is still uncertain.

We argue that neither of these initiatives draw from policy frameworks that support normative analysis of the role of citizenry. We conclude it is imperative to make visible and clear the full spectrum of meanings of “citizen science,” the contexts in which it is used, and its demands with respect to participation, engagement, and governance. In thinking holistically about participation, engagement and governance, one can consider what bearing each “citizen science” initiative has on a citizen’s rights and duties, on definitions of “societal good,” on the powers of government, and on which interests should hold sway over others.

## Abbreviations

CBPR, community-based participatory research; GPs, general practitioners; HGP, human genome project; HSCA, Health and Social Care Act; HSCIC, Health and Social Care Information Centre; ICT, information communication technology; IT, information technology; NHS, National Health Service; NIH, National Institutes of Health; PCI, “patient-centric initiatives”; PDR, “patient-driven research”; PMI, precision medicine initiative; PMI-CP, PMI-Cohort Program; TMSP, technology-mediated social participation.

## Footnotes

1 For discussion on strategies an language used by commercial firms, see [31]. For an analysis of 23andMe using Prainsack’s typology (discussed above), see [12].

2 In fact, HSCIC will not ‘anonymise’ any data; they will be coded or ‘pseudonymised.’

3 It is clear from the public response that an opt-out basis for such a project is regarded as disrespectful to the citizens. However, as one of the present authors has explained elsewhere, the care.data project is not even based on an opt-out model but amounts to conscription. See [72].

4 Apparently, in the ‘pathfinder’ areas ‘there will be lots of activity: local media work, and a new set of communication materials, including a letter sent by name to every patient, will be used to raise awareness of the programme and support GPs prior to a period of data extraction in those areas only in early 2015. ’ [73].

5 Ana Gross argues the disentangling of privacy and human agency through informed consent and anonymization initiates a process through which emerges a specific set of property rights. Research ethics itself thereby enables “the conversion of privacy into property.” When interpreted in this way, citizen science initiatives which seek to decouple the individual from his or her data to realize its value can be seen as embodying a similar ethos. In the case of government sponsored initiatives, one would have to consider the compatibility of this ethos with principles of governance [74].

6 John Rawls is one prominent example in political theory [75]. Onora O’Neil, an example in bioethics, draws heavily on autonomy as a principle, distinguishing it from individualism, and thereby establishes one possible way to think about individual interests and societal good where autonomy remains central to the thinking on governance even when informed consent is not feasible [76]. See also [77].

7 This mechanism to improve participation, or to alter the different elements of participation, and ultimately to change the role of the participant, has implications for the different types of citizen science project.
